# Adverse Reactions and Attitudes Toward the BNT162b2 COVID-19 Vaccine in Children 5 to 11 Years of Age in Japan

**DOI:** 10.2188/jea.JE20220265

**Published:** 2023-02-05

**Authors:** Naomi Matsumoto, Junya Shimizu, Yuji Yokoyama, Hirokazu Tsukahara, Takashi Yorifuji

**Affiliations:** 1 Department of Epidemiology, Faculty of Medicine, Dentistry and Pharmaceutical Sciences, Okayama University, Okayama, Japan; 2 Department of Pediatrics, National Hospital Organization, Okayama Medical Center, Okayama, Japan; 3 Department of Pediatrics, Okayama Aiiku Clinic, Okayama, Japan; 4 Department of Pediatrics, Faculty of Medicine, Dentistry and Pharmaceutical Sciences, Okayama University, Okayama, Japan

In Japan, the m-RNA vaccine to prevent novel coronavirus disease 2019 (COVID-19) for children aged 5–11 years was approved by the Ministry of Health, Labour and Welfare (MHLW) in January 2022, yet only 18% of the target children had completed the second dose as of September 12, 2022.^1^ A high incidence of mild-to-moderate adverse reactions after the m-RNA vaccination has been reported in adults, which may contribute to parents’ vaccine hesitancy for their children. International research has reported a lower frequency of systemic reactions in children after vaccination than adults,^2^^,^^3^ but few studies have been conducted on Japanese children. A post-vaccination health status survey conducted by the MHLW was small (approximately 350 subjects)^4^ and mainly covered vaccinations given at hospitals, making generalization problematic. Therefore, we conducted a large-scale survey focusing on vaccines given in clinics to ascertain the adverse reactions among Japanese children.

We targeted children aged 5 to 11 years old who received the primary doses of BNT162b2 at cooperating medical institutions (2 hospitals and 8 clinics) in Okayama Prefecture between March 11 and May 31, 2022. A flyer with a Google form QR code was distributed at the time of vaccination. Parents who agreed to receive a reminder were also encouraged to respond via e-mail approximately 7 to 10 days after vaccination. Following informed consent obtained from guardians, we surveyed the number of doses, attributes, adverse reactions among children during the first week after the primary vaccination, and parents’ attitudes toward the vaccination. We performed descriptive analyses and Poisson regression with robust variance to calculate the risk ratio of each symptom by type of attribute. Stata version 17 (StataCorp LLC, College Station, TX, USA) was used for all analyses. The study was approved by the Institutional Review Board of Okayama University Graduate School of Medicine, Dentistry, and Pharmaceutical Sciences (No. 2208-063).

Of the 1,951 children who received the first shot, 769 (39%) responded. Similarly, of the 1,819 children who received the second shot, 519 (29%) responded. We then obtained a total of 1,288 responses. Those diagnosed with COVID-19 during the study period were excluded. The sex distribution was similar, with more older children. Based on the “Underlying Diseases of Children to be Considered for Vaccination with the Novel Coronavirus Vaccine” by the Japan Pediatric Society, 3.7% (*n* = 48) of respondents had underlying medical conditions, and 45.1% (*n* = 581) had a history of allergy, such as hay fever (eTable 1). During the first week post-vaccination, the most frequently reported reactions after either dose were injection site pain and swelling, fatigue, myalgia, and headache (Table 1). Fever over 37.5°C was more frequently reported after the second dose (11.6%) than the first dose (2.7%), and none lasted longer than 4 days. The risk of post-vaccination fever was significantly higher in girls after the second vaccination (risk ratio 1.77; 95% confidential interval, 1.08–2.09), without association with age, underlying disease, or allergic history (eTable 2). The majority of parents said the reason for vaccination was to prevent infection or serious illness in their own children (eTable 3).

**Table 1.** Adverse reactions after primary dose of Comirnaty for children aged 5 to 11 years

	1st dose	2nd dose
	*n* = 769	*n* = 519
Local adverse reactions		
Pain	561 (73.0%)	337 (64.9%)
Swelling	175 (22.8%)	123 (23.7%)
Redness	79 (10.3%)	54 (10.4%)
Itchiness	66 (8.6%)	51 (9.8%)
Lymphadenopathy	7 (0.9%)	31 (6.0%)
Systemic adverse reactions		
Fever over 37.5 degrees Celsius	21 (2.7%)	60 (11.6%)
Headache	65 (8.5%)	77 (14.8%)
Fatigue	123 (16.0%)	114 (22.0%)
Chills	10 (1.3%)	12 (2.3%)
Myalgia	112 (14.6%)	63 (12.1%)
Joint pain	17 (2.2%)	24 (4.6%)
Nausea and vomiting	9 (1.2%)	9 (1.7%)
Diarrhea	10 (1.3%)	10 (1.9%)
Rash	4 (0.5%)	4 (0.8%)
Chest pain	12 (1.6%)	10 (1.9%)
Response to adverse reactions		
Antipyretic use	58 (7.5%)	68 (13.1%)
Late arrival or early leaving	5 (0.7%)	6 (1.2%)
Absence from school	36 (4.7%)	46 (8.9%)

The frequency of systemic adverse reactions in children was significantly lower than that in 8,599 healthcare workers in Okayama Prefecture, comparable to the adult official survey in Japan.^5^ The frequency of fever after the second dose was as low as 11.6% in children, approximately 1/3 of that of adults (37.5%). Similarly, the reaction frequencies of fatigue (children 22.0% versus adults 69.7%) and headache (children 14.8% versus adults 51.1%) were lower in children than in adults. Post-vaccination fever was more common in girls, consistent with adult findings.^6^ With the duty to endeavor to receive vaccination under the Immunization Act, additional investigation is needed.

## SUPPLEMENTARY MATERIAL

eTable 1. Baseline characteristics of respondents

eTable 2. Correlation between sex and post-vaccination fever

eTable 3. Reasons for deciding to have child vaccinated against COVID-19
