# Supplementary material for: Adverse Reactions and Attitudes Toward the BNT162b2 COVID-19 Vaccine in Children 5 to 11 Years of Age in Japan
Source: J Epidemiol. 2023 Feb 5;33(2):110–1. doi: 10.2188/jea.JE20220265 (PMC9794448; doi:10.2188/jea.JE20220265)
Supplement: Supplementary file 1 [file je-33-110-s001.pdf]

eTable 1. Baseline characteristics of respondents

	Respondents, No. (%)		
	All	1st dose	2nd dose
Total	1,288 (100)	769 (100)	519 (100)
Sex			
Boys	645 (50.1)	373 (48.5)	272 (52.4)
Girls	643 (49.9)	396 (51.5)	247 (47.6)
Age, years			
Age 5	105 (8.2)	63 (8.2)	42 (8.1)
Age 6	115 (8.9)	72 (9.4)	43 (8.3)
Age 7	127 (9.9)	70 (9.1)	57 (11.0)
Age 8	166 (12.9)	109 (14.2)	57 (11.0)
Age 9	198 (15.4)	116 (15.1)	82 (15.8)
Age 10	262 (20.3)	152 (19.8)	110 (21.2)
Age 11	315 (24.5)	187 (24.3)	128 (24.7)
Underlying conditions			
Any	48 (3.7)	28 (3.6)	20 (3.9)
Severe obesity	18 (1.4)	11 (1.5)	7 (1.4)
Chronic respiratory diseases (excluding bronchial asthma)	5 (0.4)	3 (0.4)	2 (0.4)
Chronic heart disease (with treatment or exercise restrictions)	2 (0.2)	1 (0.1)	1 (0.2)
Chronic renal disease (undergoing hemodialysis or peritoneal dialysis)	0 (0.0)	0 (0.0)	0 (0.0)
Neurological and neuromuscular disorders (eg, cerebral palsy, refractory epilepsy, chromosomal disorders)	19 (1.5)	10 (1.3)	9 (1.7)
Blood disorders (excluding iron deficiency anemia)	2 (0.2)	1 (0.1)	1 (0.2)
Diabetes and metabolic disorders	5 (0.4)	3 (0.4)	2 (0.4)
Malignant tumors (leukemia is included in hematologic diseases)	0 (0.0)	0 (0.0)	0 (0.0)
Rheumatic diseases, autoimmune diseases, collagen diseases	0 (0.0)	0 (0.0)	0 (0.0)
Endocrine disorders (eg, adrenal insufficiency, pituitary insufficiency, hyperthyroidism)	1 (0.1)	1 (0.1)	0 (0.0)

Digestive disorders or liver diseases (excluding normal constipation)	2 (0.2)	1 (0.1)	1 (0.2)
Suppressed immune system due to congenital immunodeficiency disorders, other diseases or treatments	2 (0.2)	1 (0.1)	1 (0.2)
Alllergic history			
Any	581 (45.1)	357 (46.4)	224 (43.2)
Bronchial asthma	130 (10.1)	78 (10.1)	52 (10.0)
Atopic dermatitis	85 (6.6)	53 (6.9)	32 (6.2)
Allergic rhinitis (including hay fever)	417 (32.4)	257 (33.4)	160 (30.8)
Food allergies	93 (7.2)	54 (7.0)	39 (7.5)
Drug allergies	7 (0.5)	5 (0.7)	2 (0.4)
Insect allergies (eg, bees)	15 (1.2)	9 (1.2)	6 (1.2)
Anaphylaxis	5 (0.4)	3 (0.4)	2 (0.4)
Other allergies	45 (3.5)	21 (2.7)	24 (4.6)
Other pre-existing medical conditions			
Previous infection	48 (3.7%)	27 (3.5%)	21 (4.1%)
Febrile convulsions	107 (8.3%)	65 (8.5%)	42 (8.1%)
Developmental disorders	111 (8.6%)	68 (8.8%)	43 (8.3%)
Kawasaki disease	26 (2.0%)	15 (2.0%)	11 (2.1%)
Vaccination site			
Hospital	41 (3.2)	23 (3.0)	18 (3.5)
Clinic	1247 (96.8)	746 (97.0)	501 (96.5)

eTable 2. Correlation between sex and post-vaccination fever

	First dose		Second dose	
	Crude	Model 1[a]	Crude	Model1[a]
	RR [95% CI]	aRR [95% CI]	RR [95% CI]	aRR [95% CI]
Female	0.86 [0.37–1.99]	0.85 [0.36–1.98]	1.77 [1.08–2.90]	1.76 [1.08–2.88]
Age		0.90 [0.74–1.09]		0.93 [0.83–1.03]
Underlying conditions		N.A.		1.24 [0.39–3.99]
Allergic history		0.59 [0.24–1.44]		1.29 [0.80–2.07]

aRR, adjusted risk ratio; CI, confidence interval; RR, risk ratio.

[a] Adjusted for age, underlying disease, and allergic history.

eTable 3. Reasons for deciding to have child vaccinated against COVID-19

	(%)
To protect the child from getting COVID-19.	83.5%
To prevent more severe illness if the child gets COVID-19.	83.0%
To prevent sequelae if the child gets COVID-19.	51.2%
To protect family and friends with underlying conditions from getting COVID-19.	31.1%
To provent spread of COVID-19 infection in preschools or schools.	56.5%
To relax mobility restrictions (eg, traveling or visiting relatives).	16.8%
Family and friends requested that the child get vaccinated	2.2%
Preschools or schools requested that the child get vaccinated	0.2%

COVID-19, novel coronavirus disease 2019.
